# A Service Audit of Psychiatric Presentations of Displaced Ukrainians in the Emergency Department or as a ward consult in University Hospital Galway from March 2022 to December 2023

**DOI:** 10.1192/j.eurpsy.2025.1233

**Published:** 2025-08-26

**Authors:** N. Goh, I. Onwuemelie, S. Patel

**Affiliations:** 1 Rehabilitation Psychiatry, National Forensic Mental Health Service; 2 Perinatal Psychiatry, Galway-Roscommon Mental Health Service; 3 Liaison Psychiatry, University Hospital Galway; 4 Liaison Psychiatry, University Hospital Portiuncula, Galway, Ireland

## Abstract

**Introduction:**

We are seeing an increasingly diverse population in the Emergency Department. There have been increasing numbers of displaced Ukrainians attending the Emergency Department and referred from the general medical/surgical wards for psychiatric assessment since the Russian invasion of Ukraine in 2022.

**Objectives:**

The objective of this retrospective audit was to examine the demographic and clinical characteristics of displaced Ukrainians presenting to the University Hospital Galway with psychiatric presentations. Our aim was to review the impact of psychiatric presentations of displaced Ukrainians on the Liaison Psychiatry/on-call Psychiatry service

**Methods:**

We utilised the Liaison Psychiatry patient database to extract data on status as a displaced Ukrainian, presenting complaint, working diagnosis, use of interpreter, location of review, outcome of review and other demographic data. This was a retrospective audit using anonymised data.

**Results:**

We found that a total of twenty-eight patient presentations were seen by Liaison Psychiatry or by the on-call Psychiatry doctor from March 2022 to December 2023 (inclusive). Twenty-three patient presentations (82.1%) were seen in the Emergency Department. Nineteen patient presentations (67.9%) required the services of an interpreter. 16 patient presentations (57.1%) were in relation to low mood with or without suicidal ideation. 16 patient presentations (57.1%) resulted in a referral to a Community Mental Health Team.

**Conclusions:**

There is a significant number of Ukrainian refugees attending the Emergency Department for psychiatric reasons. The vast majority of these patients do not speak English and require an interpreter. Being able to communicate effectively is crucial in taking a psychiatric history and further improvements should be made to improve the experience that this population of patients have in the Emergency Department. This audit can be used to inform service development in the Liaison Psychiatry department to better serve the needs of displaced Ukrainians, such as through the introduction of translated patient information documents.

**Disclosure of Interest:**

None Declared

